# Physiological and Proteomic Responses of Diploid and Tetraploid Black Locust (*Robinia pseudoacacia* L.) Subjected to Salt Stress

**DOI:** 10.3390/ijms141020299

**Published:** 2013-10-14

**Authors:** Zhiming Wang, Mingyue Wang, Likun Liu, Fanjuan Meng

**Affiliations:** 1 College of Life Science, Northeast Forestry University, Harbin 150040, China; E-Mails: 13674678261@163.com (Z.W.); 13633662163@163.com (M.W.); 2 Department of Medical Biotechnology, College of Biomedical Science, Kangwon National University, Chuncheon, Gangwon-do 200-701, Korea; E-Mail: llk_2008@126.com

**Keywords:** salt stress, *Robinia pseudoacacia* L., diploid, tetraploid, physiology, proteomics

## Abstract

Tetraploid black locust (*Robinia pseudoacacia* L.) is adaptable to salt stress. Here, we compared morphological, physiological, ultrastructural, and proteomic traits of leaves in tetraploid black locust and its diploid relatives under salt stress. The results showed that diploid (2×) plants suffered from greater negative effects than those of tetraploid (4×) plants. After salt treatment, plant growth was inhibited, photosynthesis was reduced, reactive oxygen species, malondialdehyde content, and relative electrolyte leakage increased, and defense-related enzyme activities decreased in 2× compared to those in 4×. In addition, salt stress resulted in distorted chloroplasts, swollen thylakoid membranes, accumulation of plastoglobules, and increased starch grains in 2× compared to those in 4×. However, 4× developed diverse responses under salt stress. A comparative proteomic analysis revealed that 41 and 37 proteins were differentially expressed in 2× and 4×, respectively. These proteins were mainly involved in photosynthesis, stress and defense, energy, metabolism, transcription/translation, and transportation. Distinct patterns of protein changes between 2× and 4× were analyzed. Collectively, our results suggest that the plants showed significantly different responses to salt stress based on ploidy level of the plant. The 4× possessed a better salt protection mechanism than that of 2×, suggesting salt tolerance in the polyploid plant.

## 1. Introduction

Soil salinity is a major environmental stressor that severely limits crop growth and harvest worldwide [1]. In general, salinity stress induces deleterious cellular changes, including water deficits, ion homeostasis, ionic toxicity, membrane alterations, and free radical production [2,3]. Many plants grow slowly or die under salt stress. Plants have evolved complex mechanisms to adapt to salt stress based on modifications in metabolites, gene expression and proteins. To date, many genes responding to salt stress in plants have been identified [4]. However, these genes do not offer insight into the quantity and quality of the final gene products, *i.e.*, the proteins that are regulated at the translational level [5]. Proteomics is a necessary and crucial component to genomic approaches and a powerful tool that facilitates the analysis of biochemical pathways and the complex response mechanisms of plants to the stress caused by salt, cold, and drought [6,7]. Earlier reports based on proteomics have mainly focused on the responses of diploid (2×) plants, and the proteomic knowledge of the response to salt stress by polyploids is very limited.

Polyploidy arises from the doubling of chromosomes of a single species (autopolyploidy) or the hybrids between two species (allopolyploidy). Polyploidy usually changes anatomical and morphological characteristics such as an increase in leaf thickness and pubescence [8,9]. Moreover, polyploidy also changes physiological functions or gene expression [10–12]. Accordingly, these changes may affect a lot the phenotype and the response to stress [13–18]. Due to the interest in a homogenous genetic background, some studies have tried to compare polyploids and their diploid relatives for tolerance to environmental stressors. Polyploids clearly exhibit higher tolerance to drought, heat, cold, salt, viruses, fungi, and pest stressors compared with their diploid relatives [19–22]. Therefore, polyploidy has been regarded as an efficient way to improve environmental stress tolerance in plants and has played important roles in agriculture and forestry [23]. However, the molecular and physiological basis of stress tolerance by polyploids is not well understood.

Tetraploid (4×) black locust (*Robinia pseudoacacia* L.), which is native to Korea, is a preferred tree species in the timber forest due to its rapid growth and good wood texture. Moreover, it can be used as fine feed for domestic fowl and livestock because its fleshy leaves are rich in vitamins and minerals. Importantly, tetraploid black locust is a pioneer tree species due to it wide ranging adaptability to adverse environments such as salt, drought, cold, and pests. Accordingly, tetraploid black locust has high ecological and economic value.

In this study, we report on the physiological and proteomic responses to salt stress in 2× and 4× black locust. The objectives of this study were (1) to determine growth and physiological traits under salt stress in 2× and 4× black locust (2) to improve the understanding of the molecular mechanism of differential salt tolerance in different ploidy species, and (3) to aid in the rational engineering of plants with enhanced salt tolerance.

## 2. Results and Discussion

### 2.1. Plant Growth and Physiological Response of Leaves of 2× and 4× Plants to Salt Stress

**Figure 1:**
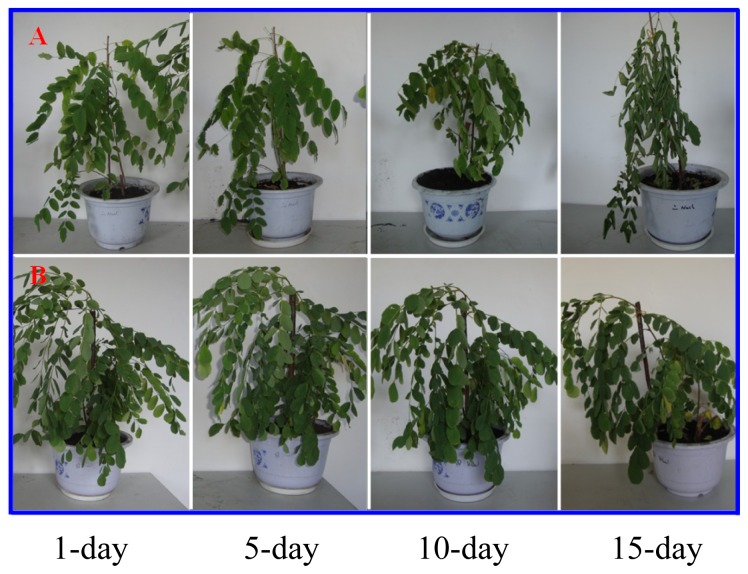
Morphological changes in *Robinia pseudoacacia* diploid (2×) and tetraploid (4×) plants growth after salt treatment (500 mM NaCl). Diploid (**A**) and tetraploid (4×) (**B**) plants were grown in a soil and sand mixture (2:1) after 1, 5, 10, and 15 days of salt treatment.

**Figure 2:**
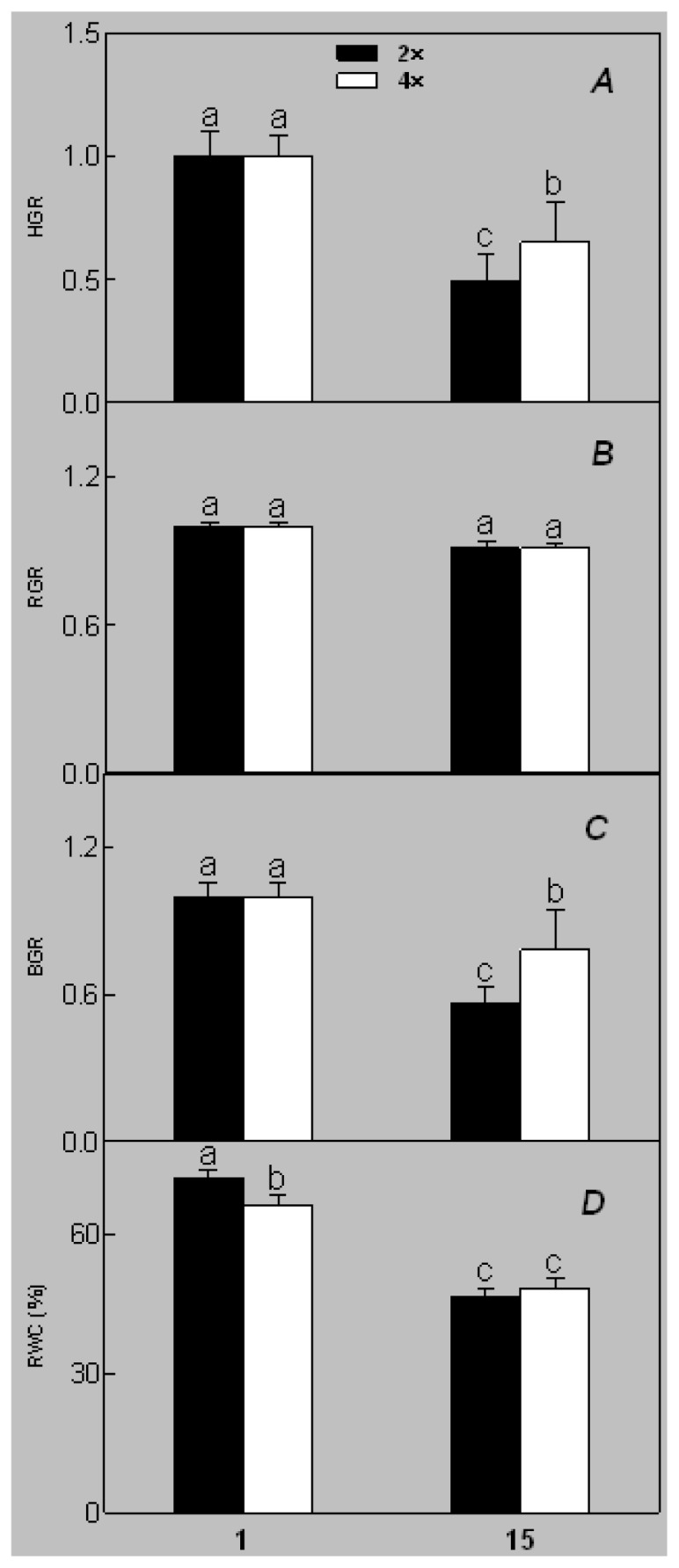
Effects of salt stress on relative height growth rate (HGR) (**A**), relative range growth rate (RGR) (**B**), relative basal diameter growth rate (BGR) (**C**), and relative water content (RWC) (**D**) of leaves in *Robinia pseudoacacia* diploid (2×) (black bars) and tetraploid (4×) (white bars) plants growing under salt stress. Values followed by different letters are significantly different from each other at *p* < 0.05 according to Duncan’s method. Each data point represents the mean ± standard error of three replicates.

Diploid plants began to wilt after 10 days and some plants died after 15 days under salt stress, as shown in Figure 1. However, 4× plants did not show significant changes even at the end of the 15 days experiment (Figure 1). In addition, salt treatment significantly inhibited plant growth (relative height growth rate (HGR) and the relative stem basal diameter growth rate (BGR)), but the difference in relative growth rate (RGR) was not significant between 2× and 4× (Figure 2A–C). In contrast, after a 10 days salt treatment, relative water content (RWC) decreased in both 2× and 4×, whereas RWC in 2× was much higher than that in 4× after 10 days of salt stress (Figure 2D).

**Figure 3:**
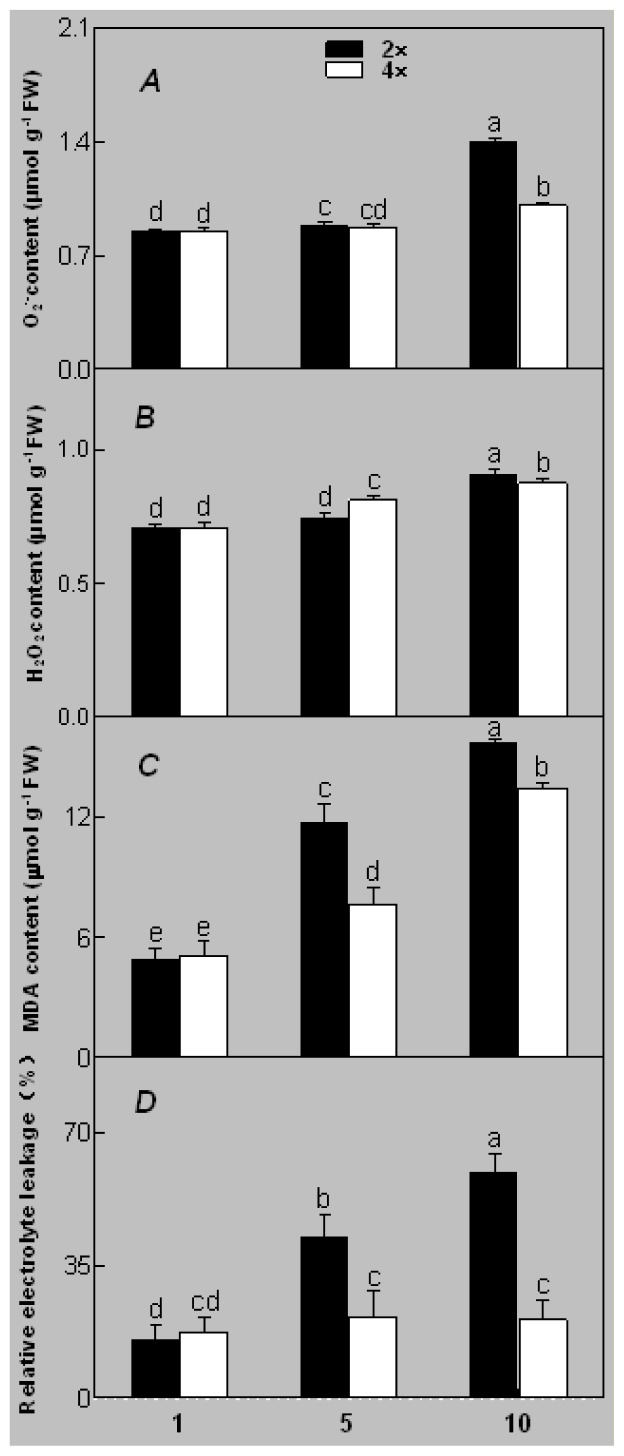
Effects of salt stress on O_2_^−^ content (**A**), H_2_O_2_ content (**B**), and malondialdehyde (MDA) content (**C**) and relative electrolyte leakage (REL) (**D**) in the leaves of *Robinia pseudoacacia* diploid (2×) (black bars) and tetraploid (4×) (white bars) plants growing under salt stress. Values followed by different letters are significantly different from each other at *p* < 0.05 according to Duncan’s method. Each data point represents the mean ± standard error of three replicates.

Salinity increased O_2_^−^ and H_2_O_2_ contents in both 2× and 4× plants (Figure 3A,B). However, 2× showed higher O_2_^−^ and H_2_O_2_ contents than did 4× at the end of 10 days of the experiment. Additionally, malondialdehyde (MDA) content and relative electrolyte leakage (REL) increased in 2× and 4× after salt treatment, and 2× showed much higher levels than that of 4× (Figure 3C,D).

**Figure 4:**
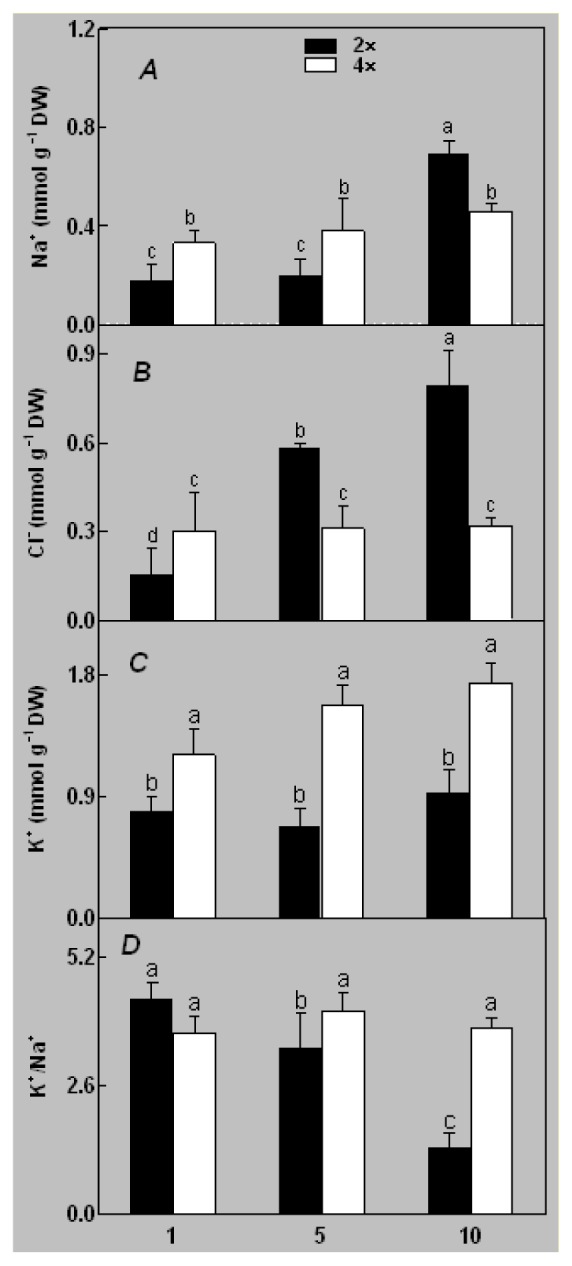
Effects of salt stress on the concentrations of Na^+^ (**A**), Cl^−^ (**B**), K^+^ (**C**), and the K^+^/Na^+^ ratio (**D**) in the leaves of *Robinia pseudoacacia* diploid (2×) (black bars) and tetraploid (4×) (white bars) plants growing after salt treatment. Values followed by different letters are significantly different from each other at *p* < 0.05 according to Duncan’s method. Each data point represents the mean ± standard error of three replicates.

At day 1 after the NaCl treatment, concentrations of Cl^−^, K^+^, and Na^+^ were higher in 4× than those in 2× plants. The K^+^/Na^+^ ratios were similar in both 2× and 4× (Figure 4A–D). After salt treatment, Na^+^, and Cl^−^ increased in 2×; however, no significant changes were detected in 4× (Figure 4A,B). In addition, NaCl treatment caused no significant changes in K^+^ concentration in 2×. However, it only caused a slight increase in the K^+^ level in 4× (Figure 4C). The K^+^/Na^+^ ratio exhibited a significant decrease in 2× when exposed to salinity but they changed little in 4× (Figure 4D).

**Figure 5:**
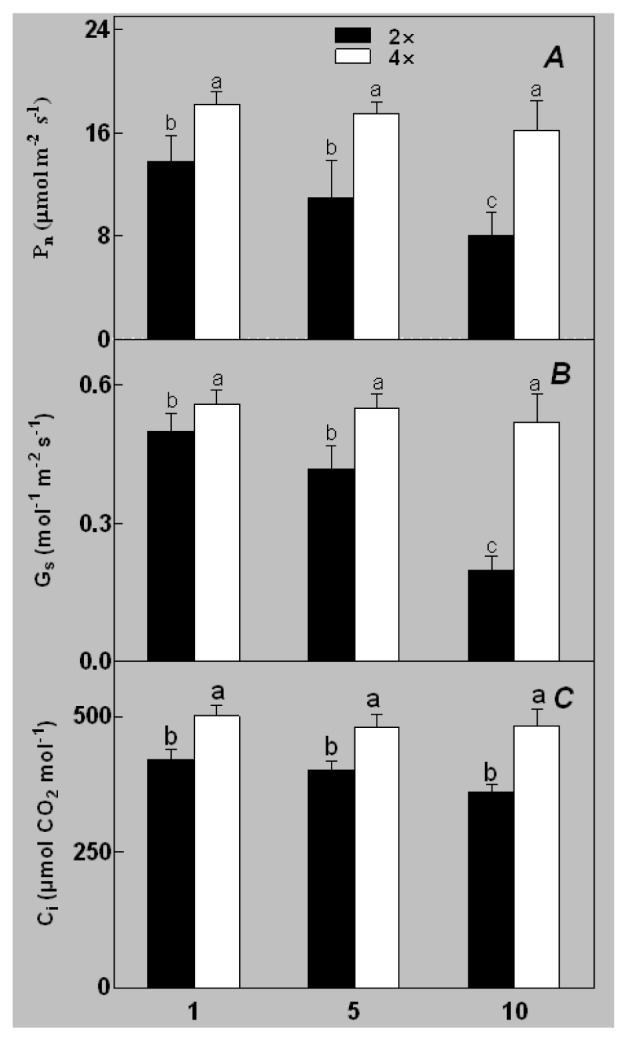
Effects of salt stress on net CO_2_ assimilation rate (*Pn*) (**A**), stomatal conductance (*Gs*) (**B**), and intercellular CO_2_ concentration (*Ci*) (**C**) in the leaves of *Robinia pseudoacacia* diploid (2×) (black bars) and tetraploid (4×) (white bars) plants growing under salt stress. Values followed by different letters are significantly different from each other at *p* < 0.05 according to Duncan’s method. Each data point represents the mean ± standard error of three replicates.

During different treatments, net CO_2_ assimilation rate (*Pn*) and stomatal conductance (*Gs*) were higher in 4× than those in 2× (Figure 5A,B). In 2×, *Pn* and *Gs* declined when plants were exposed to salinity. However, *Pn* and *Gs* in 4× plants were not significantly affected by salinity. Additionally, intercellular CO_2_ concentration (*Ci*) of 2× and 4× did not decrease significantly under salt treatment (Figure 5C).

**Figure 6:**
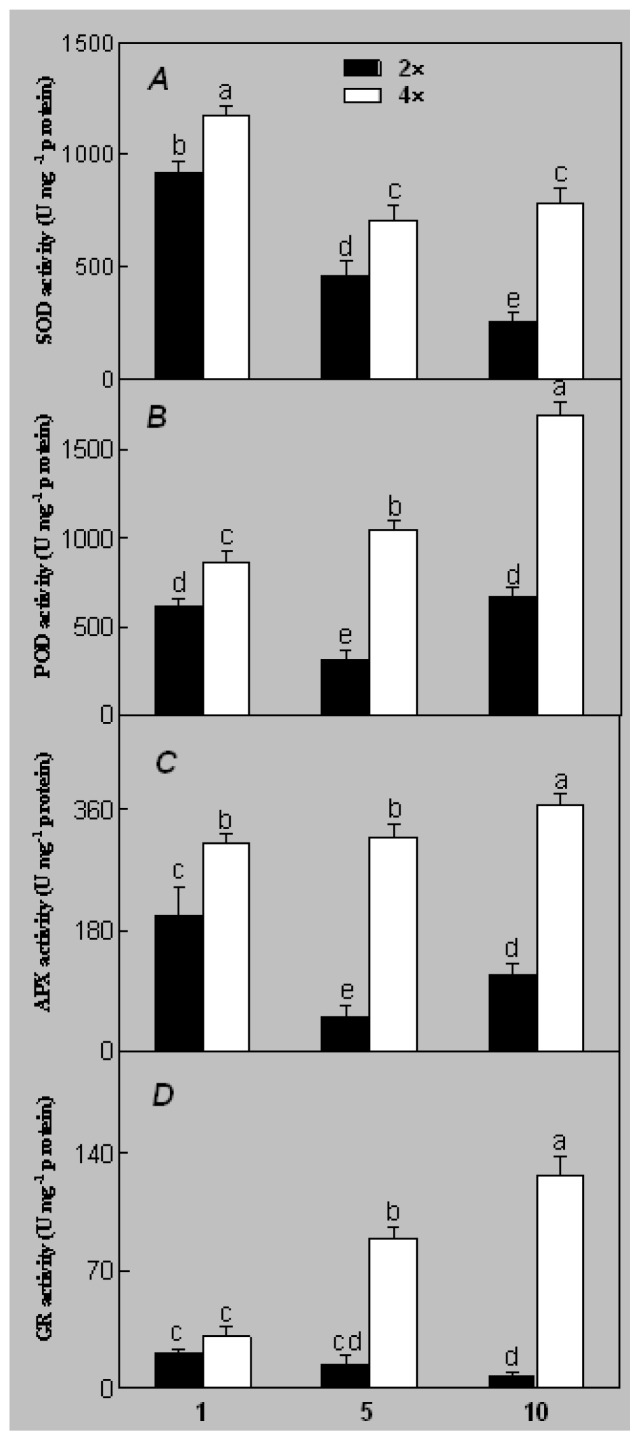
Effects of salt stress on the activities of superoxide dismutase (SOD) (**A**), peroxidase (POD) (**B**), ascorbate peroxidase (APX) (**C**), and glutathione reductase (GR) (**D**) in the leaves of *Robinia pseudoacacia* diploid (2×) (black bars) and tetraploid (4×) (white bars) plants growing after salt treatment. Values followed by different letters are significantly different from each other at *p* < 0.05 according to Duncan’s method. Each data point represents the mean ± standard error of three replicates.

Superoxide dismutase (SOD) activity decreased in 2× and 4× after salt treatment, but 4× showed higher SOD activity than that of 2× (Figure 6A). The activities of peroxidase (POD), ascorbate peroxidase (APX), and glutathione reductase (GR) increased under salt stress in 4× but not in 2× (Figure 6B–D). In particular, 4× plants showed significantly higher APX and GR activities than those of 2× when subjected to salinity (Figure 6C,D).

**Figure 7:**
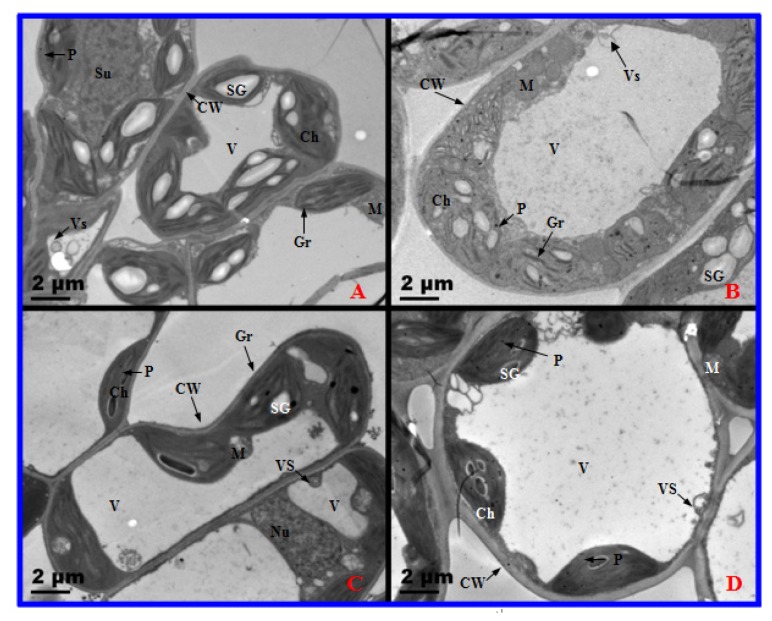
Transmission electron micrographs of diploid (2×) and tetraploid (4×) *Robinia pseudoacacia* mesophyll cells after one day and 10 days of salt treatment. (**A**) 0 day, 2×; (**B**) 0 day, 4×; (**C**) 10 days, 2×; (**D**) 10 days, 4×. CW, cell wall; Ch, chloroplast; M, mitochondrion; Nu, nucleolus; P, plastoglobule; SG, starch granule; Gr, granum; V, vacuole; Vs, small vesicle.

**Figure 8:**
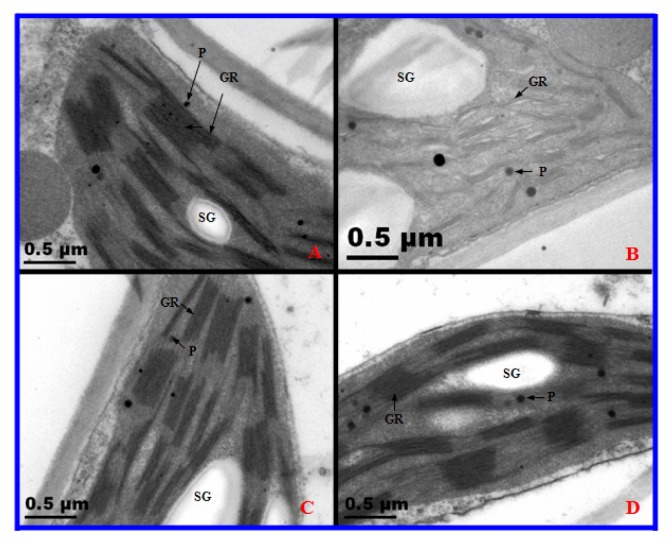
Transmission electron microscopy observations of chloroplasts in diploid (2×) and tetraploid (4×) *Robinia pseudoacacia* after one day and 10 days of salt treatment. (**A**) 0 day, 2×; (**B**) 10 days, 2×; (**C**) 0 day, 4×; (**D**) 10 days, 4×. Gr, granum; thylakoid (Th); P, plastoglobule; SG, starch grain.

The results for the physiological responses of plant growth to salt stress were different (Figures 1–8). Salt stress inhibited plant growth and decreased net photosynthetic rate and stomatal conductance in 2×. However, the decreases in these parameters were less in 4× than those in 2×. These results agree with our earlier research showing that 4× plants are relatively salt-tolerant compared to that of 2× plants [24]. Similar results were reported by another group [25]. In Figure 5, our results showed that *Pn* and *Gs* for 4× were not sensitive in response to salt stress after RWC decreases are detectable. It may be a reason that the salt stress caused less pronounced inhibition of photosynthesis in 4× than in 2×. In other words, 4× plants have higher water use efficiency under salt stress. Thus, 4× can keep photosynthesis stability under low RWC to adapt salt stress. At the same time, RWC is not only factor influencing *Pn*. The decrease in growth and stomatal conductance may result from reduced leaf water content. *Gs* plays an important role in the change in *Pn*. Little change in *Ci* was observed compared with that of *Gs* (Figure 3). This indicates that photosynthesis under salt stress may be affected by other factors such as the availability of ATP and Rubisco activity [26]. The changes between *Pn*, *Gs*, and *Ci* in 2× and 4× plants were different, which was further supported by the proteomics results. The expression levels of 19 and 16 proteins including Rubisco large subunit, rubisco activase and ATP synthase CF1 alpha subunit increased in 2× and 4× after salt treatment, respectively. MDA and REL are important indicators of the damage caused by salt stress [27]. The present results show that salinity significantly increased MDA content and REL in 2× more than those in 4×, indicating more damage to membranes in 2× plants.

Salt stress induces the accumulation of reactive oxygen species (ROS) such as H_2_O_2_ and O_2_^−^. Excess accumulation of ROS causes oxidative damage to membrane lipids, nucleic acids, and proteins [28]. H_2_O_2_ and O_2_^−^ accumulated in leaves of 2× and 4× plants after salt treatment. In general, plants reduce or scavenge ROS through antioxidant enzymes such as SOD, POD, APX, and GR. Interestingly, the activities of the antioxidant enzymes (POD, APX and GR) in 4× plants increased under salt stress. All antioxidant enzymes play an important role adjusting the cellular redox state [29]. In contrast, these enzymes (POD, APX and GR) decreased in 2×. Antioxidant enzyme activities increase in most salt-tolerant plants [30]. However, SOD activity declined gradually in both 2× and 4× during salt treatment. Conversely, 4× plants showed higher SOD activity than that of 2×, indicating that 4× has a more efficient enzymatic antioxidant system against salt stress and adjusting to ROS than those of 2× plants.

### 2.2. Ultrastructural Responses of 2× and 4× Plants to Salt Stress

The ultrastructure of the mesophyll cells of both 2× and 4× plants were similar after one day of salt treatment (Figure 7A,C). No significant differences were found in the chloroplasts (*Cs*)/thylakoid (Th) of the mesophyll cells of 2× and 4× plants after one day of salt treatment (Figure 8A,C). Nevertheless, the ultrastructure of 2× mesophyll cells changed noticeably after 10 days of salt treatment (Figure 7B). In 2×, stress caused by severe salinity resulted in distorted Cs, swollen Th, and an accumulation in plastoglobules after 10 days of salt treatment. In addition, the number of starch grains increased (Figure 8B). However, the ultrastructural morphological injuries were not apparent in 4× salt-stressed plants (Figures 7D and 8D).

Salinity stress affects cell ultrastructure in many plant species. Photosynthesis occurs in chloroplasts; hence, it is the most severely impacted organelle under salt stress. Salinity induces severe disorganization in chloroplasts, leading to distorted chloroplasts and dilated thylakoid membranes in mesophyll cells of *A. littoralis* [31]. However, in this study, chloroplasts of 2× became swollen, plastoglobules and starch granules accumulated, and thylakoids exhibited disorganization after salt treatment (Figures 7 and 8). In addition, 2× plants showed a decline in *Pn*, *Gs*, *Ci*, growth rate, and an increase in REL. These results reveal that salt stress destroyed the structure and function of mesophyll cells. Some reports have shown that disorganized chloroplasts are the main source of reactive oxygen and are involved in leaf senescence [32]. Therefore, 2× displayed accelerated senescence under salt stress. However, less serious injury was observed in chloroplasts of salt-stressed 4× than that in salt-stressed 2× at the same treatment stage, which again suggests that 2× suffered from greater negative effects than that of 4× when grown under high salinity conditions. Therefore, 4× can keep stable photosynthesis by organized cell ultrastructure.

### 2.3. Identification of Differentially Expressed Proteins after Salt Treatment by Two-Dimensional Electrophoresis (2-DE)

**Figure 9:**
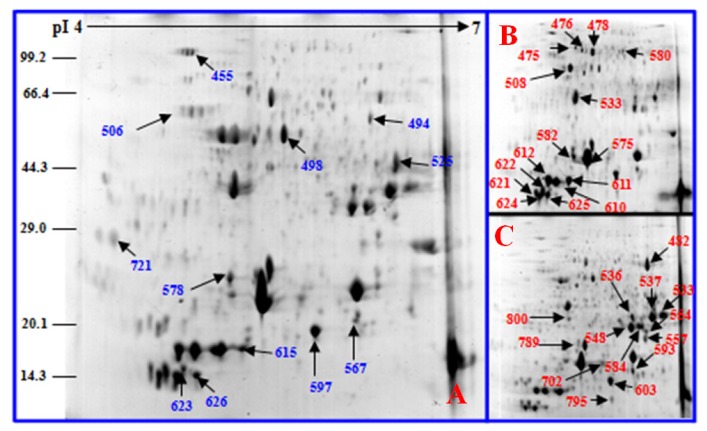
Coomassie Brilliant Blue (CBB)-stained two-dimensional electrophoresis gels of proteins from *Robinia pseudoacacia* diploid (2×) leaves. Proteins were separated on a 13 cm IPG strip (pH 4–7 linear gradient) using isoelectric focusing, followed by sodium dodecyl sulfate polyacrylamide gel electrophoresis on a 12.5% gel. (**A**) 1-day NaCl-treated leaves; (**B**) 5-day NaCl-treated leaves; (**C**) 10-day NaCl-treated leaves. Blue and red numbers indicate proteins that increased and decreased between control and stressed samples, respectively. The proteins are listed in Table 1.

**Figure 10:**
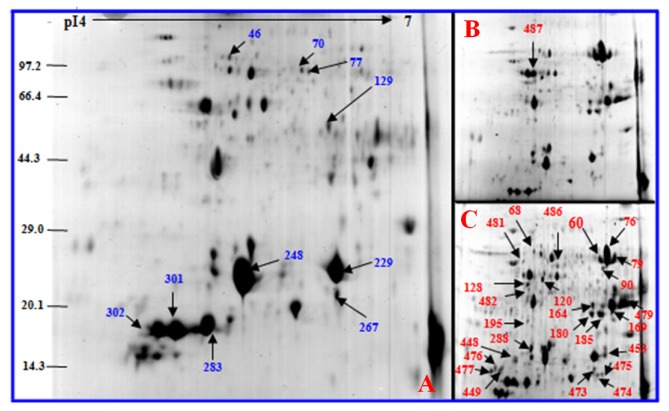
Coomassie Brilliant Blue (CBB)-stained two-dimensional electrophoresis gels of proteins from *Robinia pseudoacacia* tetraploid (4×) leaves. Proteins were separated on 13 cm IPG strip (pH 4–7 linear gradient) using isoelectric focusing, followed by sodium dodecyl sulfate polyacrylamide gel electrophoresis on a 12.5% gel. (**A**) 1-day NaCl-treated leaves; (**B**) 5-day NaCl-treated leaves; (**C**) 10-day NaCl-treated leaves. Blue and red numbers indicate proteins that increased and decreased between control and stressed samples, respectively. The proteins are listed in Table 2.

To investigate the changes in protein profiles under salt stress, total protein from the leaves of control and salt-treated plants was extracted and analyzed by 2-DE. Approximately 800 protein spots were detected in 2× and 4× plants, respectively. Master 2-D gel maps are shown in Figures 9 and 10. All protein spots were quantitatively analyzed, but only the protein spots that showed two-fold or more expression (*p* < 0.05) changes under the salt treatments were submitted for protein identification. All 41 protein spots from 2× gels and 37 protein spots from 4× gels were detected as differentially expressed spots (Figures 9 and 10; Tables 1 and 2). Twenty-eight proteins were up-regulated in 2× plants. Of which 13 proteins increased gradually in abundance in salt-treated plants, whereas 13 proteins increased in expression after 5 days of salt treatment and then decreased after 10 day of salt treatment. Two proteins decreased and then increased under salt stress compared to that in controls. Twenty-five proteins were up-regulated in 4× plants. Among them, 21 proteins increased gradually compared to controls and only four increased and then decreased with salt treatment.

**Figure 11:**
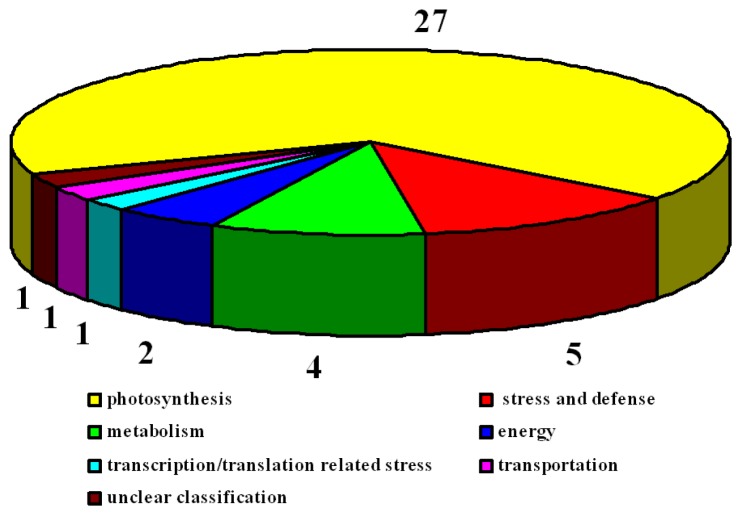
Functional categorization of proteins in *Robinia pseudoacacia* diploid (2×) plants under salt stress. Digits indicate the protein number of each functional category.

**Figure 12:**
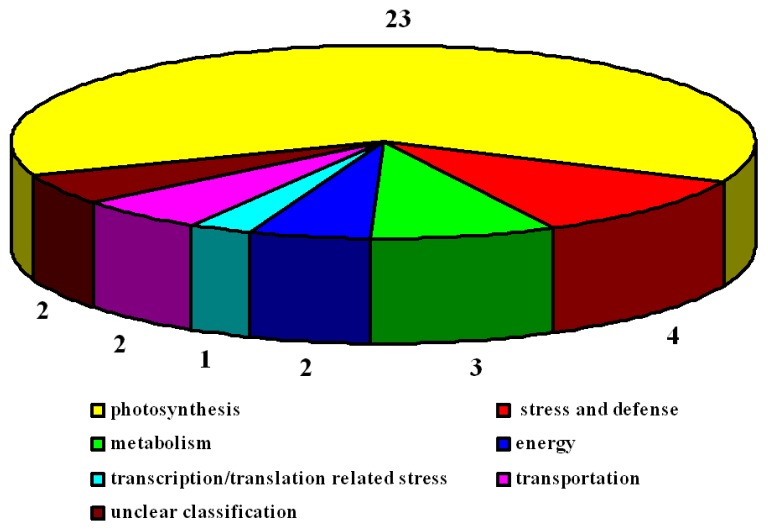
Functional categorization of proteins in *R. pseudoacacia* tetraploid (4×) plants under salt stress. Digits indicate the protein number of each functional category.

To further examine the differentially expressed proteins, all 78 protein spots were excised, digested, and submitted for protein identification. Based on the data from BLASTP, Gene Ontology, and some literature, the identified proteins covered a wide range of molecular functions (Tables 1 and 2). In 2×, 41 identified proteins were classified into seven categories (Figure 11). The largest group of proteins was associated with photosynthesis (27), followed by stress and defense (5), metabolism (4), energy (2), transcription/translation related (1), transportation (1), and unclear classification (1). In 4×, 39 identified proteins were also classified into seven groups (Figure 12). Most of the proteins were involved in photosynthesis (23), followed by stress and defense (4), metabolism (3), energy (2), transportation (2), transcription/translation related (1), and unclear classification (2).

#### 2.3.1. Photosynthesis-Related Proteins

Salt stress is a major environmental factor that limits the efficiency of photosynthesis. In our study, nearly half the proteins (27 in 2× and 23 in 4×) were photosynthesis-related proteins. Among them, a number of proteins (20 in 2× and 17 in 4×) were assigned to the Rubisco large subunit from different plant species but are visualized at positions with very different pIs and molecular weights (Tables 1 and 2). There results were not surprising because Rubisco is the most abundant protein in leaves. In addition, proteins in multiple spots can be translated from alternatively spliced mRNAs [33,34]. Two ribulose-bisphosphate carboxylase were only detectable in the treated 2×, and three Rubisco activases were only detectable in the treated 4×, suggesting that they responded specifically to salt stress. Among them, the two Rubisco activases increased in 4× after salt treatment. Previous reports also showed that the main role of this activase as an ATPase protein is maintenance of the catalytic activity of Rubisco by removing inhibitory sugars from the active site of uncarbamylated and carbamylated Rubisco [35,36]. Thus, increased Rubisico activase activity may have lead to increased photosynthetic rate (*Pn*) and increased growth rate. This result substantiated that 4× can tolerate high salt stress.

Photosystem II (PSII), as the core complex in photosynthesis, mediates oxygen evolution activity. PSII proteins (spot 578) in 2× were down-regulated under salt stress, suggesting that PSII activity was inhibited. These results were similar to previous observations [37]. In addition, two ribulose-bisphosphate carboxylase (RuBPCase) were down-regulated after salt treatment. RuBPCase is responsible for the primary step in CO_2_ fixation. Down-regulation of RuBPCase leads to a reduction in photosynthetic capacity. But, changes in these enzymes were not identified in 4× under salt stress. A possible reason is that 2× and 4× used different strategies to combat salt stress. Particularly, ATP synthase CF1 alpha subunit showed increased levels in 4× but decreased levels in 2× at 10 days of salt treatment. The increase in ATP synthase CF1 alpha subunit increases photosynthesis rate and down-regulates the Calvin cycle enzyme phosphoglycerate kinase [38]. Clearly, 4× had a greater capability to tackle salt stress by accumulating more photosynthesis-related proteins.

#### 2.3.2. Stress and Defense Proteins

Salt stress can lead to ion imbalance, hyperosmotic stress, and oxidative damage. Thus, plants induce various stress response proteins, many of which are crucial components of the plants self-defense network. Five and four proteins had altered translation levels in response to salinity stress in 2× and 4×, respectively. Here, they were only visible in the treated 2× and 4× plants. Agglutinin I polypeptide B and phenylalanine ammonia lyase were significantly up-regulated after salt treatment in 2×, whereas heat shock protein 70 and plastidic aldolase decreased under salt stress (Table 1). In 4×, four proteins involved in the defense reaction, including legume lectin of the bark, sedoheptulose-1,7-bisphosphatase, phenylalanine ammonia lyase, and APX remarkably accumulated under salt stress (Table 2). The accumulation of proteins associated with defense could help 4× survive the high salt conditions. Our previous study showed that 4× plants have higher APX activity than that of 2× under salt stress. APX can detoxify H_2_O_2_ to H_2_O and plays an important role resisting salt stress in plants [39]. These results were not surprising because APX plays an important role in salt stress tolerance.

Lectins are carbohydrate-binding proteins expressed in plants upon exposure to biotic or abiotic stressors such as drought, salinity, heat, hormone treatment, pathogen attack, or insect herbivory. In the present study, the abundance of agglutinin I polypeptide B in 2× and chain A, a legume lectin of the bark in 4×, increased after salt treatment. Lectins reduce the detrimental effects of salinity-induced oxidative stress in wheat seedlings, which is consistent with other findings [40]. Clearly, this suggests a very important role of lectins in plant salt tolerance.

#### 2.3.3. Energy Proteins

When plants are exposed to high levels of salt, extra energy may be consumed to prevent damage. Phosphoglycerate kinase (PGK) and mitochondrial F1-ATPase beta subunits increased in 4× under salinity stress (Table 2). Some reports have revealed that PGK levels are enhanced by various stress conditions in several species [41]. PGK catalyzes the formation of ATP to ADP, which is essential for carbon fixation in plants. Enhancement of PGK would provide more ATP and ensure sufficient energy for plants to resist salt stress. In addition, the mitochondrial F1-ATPase beta subunit was up-regulated in 4× by salt treatment. Such up-regulation in the of F1-ATPase beta subunit protein level in response to salt treatment is likely to affect the tricarboxylic acid cycle, electron transport, and increase ATP synthesis. This may be one of the important mechanisms by which salt treatment reduces the extent of salinity-induced oxidative stress.

The same PGK (spot 494) was down-regulated in 2× under salt stress. However, another PGK was up-regulated. This was inconsistent with what was found in 4×. This different response to salt stress between the two plants is interesting. However, the PGK protein was found in two spots, which is presumably due to post-transcriptional modification or proteolytic degradation of proteins *in vivo* and *in vitro.*

#### 2.3.4. Metabolic Proteins

Aconitate hydratase and pyruvate kinase are essential respiratory metabolism enzymes in plants. The abundance of aconitate hydratase and pyruvate kinase suggests that the tricarboxylic acid (TCA) and glycolysis increase under salinity stress. Enhancement of TCA and glycolysis would produce more ATP. Increases in aconitate hydratase and pyruvate kinase involved in respiratory metabolism showed the capacity of 2× plants to recover from salt stress. In contrast, no proteins associated with TCA or glycolysis changed significantly under salt stress in 4× plants. Besides, we found that the levels of chalcone synthase (CHS) and enolase changed remarkably in 4× under salt stress. A large number of investigations have shown that CHS and enolase are induced by various environmental stressors. Therefore, it was considered that 4× plants used another pathway to resist salt stress, or 4× as a halophyte had a high capacity to prevent damage resulting from oxidative stress.

In this study, to keep genetic stability, we propagated plants by cutting. Generally, cutting propagation can produce genetically identical progeny compared with hybrids propagation. Thus, cutting reproduction strongly influences genetic variation. Now, other methods such as flow cytometry and chromosome counting have been used for ploidy estimation (11).

## 3. Experimental Section

### 3.1. Plant Materials and Growth Conditions

All materials are introduced directly from South Korea to China by Beijing Forestry University. The diploid and tetraploid plants are from a germplasm. Thus, they have the same genetic origin. Thirty uniform plants (2-years-old) from 2× and 4× black locust were collected from Beijing Forestry University in Beijing, China and planted in plastic pots (18 cm in diameter and 18 cm in depth) filled with 2:1 (*v*/*v*) mixture of soil and sand. The experiments were carried out at Harbin Experimental Forest Farm of Northeast Forestry University in June 2011. Potted plants were grown in the greenhouse (day/night air temperature, 28/22 °C, photoperiod 12 h, and approximately 65%–85% relative humidity) for 1 month. Previous research showed that 4× tolerate high salt stress (500 mM NaCl) for a short time. Thus, in this study, we treated plants with 500 mM NaCl for 15 days. After 1, 5, 10, and 15 days of treatment, the leaves were harvested, immediately frozen in liquid nitrogen, and stored at −80 °C prior to physiological and proteomic experiments. At least three independent biological experiments for each treatment were replicated.

### 3.2. Morphological and Biomass Measurements

The morphological traits of all plants were observed and photographed at 1, 5, 10, and 15 days of treatment. At the beginning and end of the salt treatments, height, range, and stem basal diameter of plants and leaf water content were recorded. The HGR, RGR, and the relative growth rate of stem basal diameter (BGR) were calculated using the following formulas: HGR = (*h′_2_* − *h′_1_*)/(*h_2_* − *h_1_*), where *h′_1_* is the height of salt-stressed plant at the beginning, *h′_2_* is the height of salt-stressed plants at the end, *h_1_* is the height of control plants at the beginning, *h_2_* is the height of control plant at the end. RGR = (*r′_2_* − *r′_1_*)/(*r_2_* − *r_1_*), where *r′_1_* is the range of stressed plants at the beginning, *r′_2_* is the range of stressed plants at the end, *r_1_* is the range of control plants at the beginning, *r_2_* is the range of control plants at the end. BGR = (*b′_2_* − *b′_1_*)/(*b_2_* − *b_1_*), where *b′_1_* is the stem basal diameter of stressed plants at the beginning, *b′_2_* is the stem basal diameter of stressed plants at the end, *b_1_* is the stem basal diameter of control plants at the beginning, *b_2_* is the stem basal diameter of control plants at the end. At 9:30 AM, the fully expanded leaves were collected to measure relative water content (RWC) of leaves using the following formula: RWC (%) = (FW − DW)/(TW − DW) × 100, where FW is fresh weight, TW is turgid weight after rehydrating the samples for 24 h, and DW is dry weight after oven-drying the samples at 85 °C for 24 h.

### 3.3. Ion Content Analysis

Leaves were dried at 80 °C and digested in nitric acid (1% HNO_3_ (*v*/*v*)) using the method of Wolf (1982) to measure Na^+^ and K^+^[42]. Na^+^ and K^+^ were analyzed by flame emission using atomic absorption spectrophotometry (PerkinElmer Analyst 800, Waltham, MA, USA). Cl^−^ was quantified by a modified silver titration method described by Chen *et al.* (2001) [43].

### 3.4. Determination of Superoxide Radical, Hydrogen Peroxide, Lipid Peroxidation and Relative Electrolyte Leakage

Leaves (0.5 g) were ground at 4 °C and then homogenized in 5 mL 50 mM potassium phosphate buffer (pH 7.8) containing 0.1 mM EDTA, 1% (*w*/*v*) PVP, 0.1 mM PMSF, and 0.2% (*v*/*v*) Triton X-100. The mixture was centrifuged at 12,000× *g* for 15 min at 4 °C. One millilitre the supernatant was mixed with a mixture of 1 mL hydroxylamine hydrochloride for 1 h, 1 mL β-aminobenzene sulfonic acid, and 1 mL α-naphthylamine, and then the solution was incubated at 25 °C for 20 min. The concentration of superoxide radical was determined by measuring the absorbance of the mixture at 530 nm using a NaNO_2_ standard curve. Hydrogen peroxide (H_2_O_2_) content was determined by measuring the absorbance of the H_2_O_2_ titanium complex at 410 nm using known concentrations of H_2_O_2_ as the standard curve. Lipid peroxidation was estimated as MDA content using a modified method. Leaves (0.2 g) were homogenized in 5 mL of 5% of trichloroacetic acid (TCA) and centrifuged at 12,000× *g* for 15 min. Two ml of supernatant was added to a test tube containing the mixture of 2 mL of 20% TCA, 0.01% butylated hydroxytoluene, and 0.6% thiobarbituric acid. The mixture was heated in boiling water for 30 min, and then quickly cooled on ice. After centrifugation at 12,000× *g* for 10 min, the absorbance of the supernatant was determined at 450, 532 and 600 nm.

Twenty leaf discs (1.0 cm^2^) from the third to fifth fully expanded leaves were vacuum-infiltrated in 10 mL deionized water for 30 min and maintained in water for 6 h. Using a portable conductivity detector (LC116, Mettler Toledo Instruments Co., Ltd., Shanghai, China), the conductivity of the bathing solution (C1) was determined. Then, the leaf discs and the bathing solution were boiled for 10 min and thoroughly cooled to room temperature, and the conductivity of the resulting solution (C2) was determined. The REL was calculated (REL (%) = (C1/C2) × 100).

### 3.5. Photosynthesis Analysis

Net photosynthetic rate (*Pn*), *Gs*, and intercellular CO_2_ concentration (*Ci*) were measured at 0, 5, and 10 days of salt treatment from 9:00 to 11:30 in the morning with a portable photosynthesis measuring system LI-COR 6400 (LI-COR Inc., Lincoln, NE, USA).

### 3.6. Antioxidant Enzyme Activity Analysis

SOD (EC1.15.1.1) activity was measured following the method of Roth and Gilbert (1984) [44]. The reaction mixture contained 20 μL enzyme extract, 50 mM sodium phosphate buffer (pH 7.8), 100 μM EDTA, and 10 mM pyrogallol. Enzyme activity was detected at 420 nm by spectrophotometer. POD (EC1.11.1.7) activity was measured according to the method of Nickel and Cunningham (1969) [45]. The reaction mixture contained 25 mM PBS (pH 7.0), 0.05% guaiacol, 10 mM H_2_O_2_, and enzyme extract. POD activity was measured at 470 nm. GR (EC1.6.4.2) activity was assayed using the method of Nordhoff *et al.* (1993) [46]. GR activity was determined by NADPH oxidation at 340 nm. The reaction mixture contained 10 μL enzyme extract, 100 mM potassium phosphate buffer (pH 7.8), 0.2 mM NADPH, 2 mM EDTA, and 0.5 mM glutathione. The reaction was initiated by adding NADPH at 25 °C. The APX (EC1.11.1.11) activity assay was carried out using the method of Nakano and Asada (1981) [47]. The reaction mixture contained 50 mM sodium phosphate buffer (pH 7) including 0.2 mM EDTA, 0.5 mM ascorbic acid, 50 mg of BSA, and crude enzyme extract. The reaction was started by adding H_2_O_2_ at a final concentration of 0.1 mM.

### 3.7. Ultrastructural Leaf Analysis

Fresh leaf segments (about 1.5 cm in length and 0.5 cm in width) were fixed in 2.5% glutaral pentanedial (*v*/*v*) at 4 °C for 2 h, washed twice in 0.1 M PBS (sodium phosphate buffer, pH 6.8) at 4 °C. Then, they were postfixed in 2% osmium tetraoxide (O_s_O_4_) for 2 h, sequentially dehydrated in 50%, 70%, 90%, and 100% acetone, and embedded in Epon 812 for 2 h. Ultra-thin sections (70 nm) were sliced, stained with uranyl acetate and lead citrate, and then mounted on copper grids for viewing on the H-600 IV TEM (Hitachi, Tokyo, Japan) at an accelerating voltage of 60 kV.

### 3.8. Extraction of Total Leaf Protein

Total leaf protein of plants at different treatment times (0, 5, and 10 days) was extracted using the method of Hurkman and Tanaka (1986) [48]. Fresh leaf samples were ground in liquid nitrogen using a mortar and pestle to make a fine powder, and then suspended in 10% ice-cold TCA in acetone containing 0.07% β-mercaptoethanol. The suspension was allowed to precipitate overnight at −20 °C and was centrifuged at 15,000× *g* for 50 min at 4 °C. The supernatant was removed, the precipitate was washed three times with ice-cold acetone containing 0.07% β-mercaptoethanol for 1 h at −20 °C, and the protein pellet was air-dried and stored at −80 °C. The protein powder was solubilized in lysis buffer (7 M urea, 2 M thiourea, 4% *w*/*v* CHAPS, 40 mM DTT, 2% *v*/*v* pH 4–7 IPG buffer, and 4% *w*/*v* PMSF), and the supernatant was collected by centrifugation at 15,000× *g* for 50 min at 4 °C. The protein content was assayed using bovine serum albumin as the standard according to the method of Bradford (1976) [49].

### 3.9. 2-DE Image Analysis and Gel Staining

2-DE was carried out according to the method of Bjellqvist *et al.* (1982). A mixture of 300 μg protein sample in 350 μL of rehydration buffer containing 7 M urea, 2 M thiourea, 2% *w*/*v* CHAPS, 40 mM DTT, and 0.5% *v*/*v* IPG buffers, pH 4–7 (GE Healthcare Bio-Sciences Corp., Piscataway, NJ, USA), and 0.01% bromophenol blue was prepared. The mixture was loaded onto IPG strips (13 cm, linear pH 4–7, GE Healthcare Bio-Sciences, Uppsala, Sweden). After overnight rehydration of the IPG strips at 20 °C, isoelectric focusing was carried out on an Ettan IPGphorII (GE Healthcare, Bio-Sciences, Uppsala, Sweden) at 20 °C. Isoelectric focusing was performed using the following procedure: 30 V for 2 h, 100 V for 1 h, 500 V for 1 h, 1000 V for 1 h, gradient 8000 V for 0.5 h, and 8,000 V rapid focus for 6 h. After focusing, the strips were equilibrated by reduction with DTT and carboxymethylation with iodoacetamide. The second dimension was performed on 12.5% (*w*/*v*) polyacrylamide gels at 15 mA per gel for 30 min followed by 30 mA until the bromophenol blue ran off the bottom of the gel. Gels were stained with Coomassie Brilliant Blue R-250 and destained the next day.

Gel images were scanned using an ImageScanner III (GE Healthcare, Bio-Sciences, Uppsala, Sweden). Images were analyzed with ImageMaster 2D Platinum 7.0 software (Amersham Biosciences, Piscataway, NJ, USA, 2011). The average volume percent values were calculated from three technical replicates to represent the final volume percent values of each biological replicate. The experimental molecular mass and PI of the protein spots were determined by 2-DE standards and interpolation of missing values on the IPG strips. Spots were quantified based on total density of the gels by the percentage volume. Significantly different spots, which were determined as *p* < 0.05 and a change of more than two-fold in abundance, were considered to be differentially accumulated proteins, and they had to be consistently present in three replications.

### 3.10. In-Gel Digestion and Matrix-Assisted Time of Flight Mass Spectroscopy (MALDI-TOF-MS) Analysis

Selected protein spots were excised, washed with 50% (*v*/*v*) acetonitrile in 0.1 M NH_4_HCO_3_, and dried at room temperature. Proteins were reduced with 1 mM DTT and 2 mM NH_4_HCO_3_ at 55 °C for 1 h and alkylated with 55 mM iodoacetamide in 25 mM NH_4_HCO_3_ in the dark at room temperature for 45 min. The gel pieces were thoroughly washed with 25 mM NH_4_HCO_3_, 50% ACN, 100% ACN, and dried. The proteins were digested in 10 mL modified trypsin (Promega, Madison, WI, USA) solution (1 ng mL^−1^ in 25 mM ammonium bicarbonate) during an overnight incubation at 37 °C. Digests were immediately spotted onto 600 mm Anchorchips (Bruker Daltonics, Bremen, Germany). Spotting was achieved by pipetting 1 mL of analyte onto the MALDI target plate in duplicate and then adding 0.05 mL of 20 mg mL^−1^ α-CHCA in 0.1% TFA/33% (*v*/*v*) ACN, which contained 2 mM ammonium phosphate. All samples were analyzed in the positive-ion reflectron mode on a TOF Ultraflex II mass spectrometer (Bruker Daltonics, Billerica, United states). Each acquired mass spectra (*m*/*z* range 700–4000, resolution 15,000–20,000) was processed using FlexAnalysis v2.4 software (Bruker Daltonics, Bremen, Gemeny, 2004). Proteins were identified with Mascot software (http://www.matrixscience.com) based on the mass signals to search for proteins in the SwissProt, NCBInr, and MSDB databases.

### 3.11. Statistical Analyses

Statistical analyses were performed with SPSS 17.0 software (SPSS Inc., Chicago, IL, USA, 2009). All parameters are presented as mean ± standard error and were obtained from at least three replicates and analyzed using Duncan’s multiple range test or Student’s *t*-test. A *p*-value < 0.05 was considered significant.

## 4. Conclusions

Tetraploid black locust has high ecological and economic value. Until now, little was known about the implications of ploidy level in black locust on the physiological and proteomic responses under salt stress. In this study, the physiological and proteomic responses of 2× and 4× black locust were detected under salt stress during different times. Our results demonstrated that 2× plants suffered from greater negative effects than those of 4× plants according to their morphological and physiological characteristics under salt stress. In addition, 2-DE was used to analyze ploidy differences of the black locust leaf proteome under salt stress. Therefore, 4× plants have higher levels of several ROS scavenging enzymes and accumulate photosynthesis-related enzymes, defense protein and energy proteins to cope with salt stress compared with 2×. These results contribute to selection of some specific proteins during ploidy process. In addition, the results suggested that 4× had a greater capability to defend against salt stress by accumulating more related proteins. Our results provide more information to further understand the molecular and physiological basis of stress tolerance in polyploid plants.

## Figures and Tables

**Table 1:** Protein identities and their relative changes in leaves of tetraploid *Robinia pseudoacacia* after salt stress.

Spot No. a	Protein Name	Species	gi Number b	Theoretical MW(Da)/pI c	Experimental MW(Da)/pI d	Score e	M f	C (%) g	V% ± SE h (1/5/10 days)
**Energy**									
494	phosphoglycerate kinase	*N. benthamiana*	313585890	50.05/7.66	58.00/6.34	172	10	19	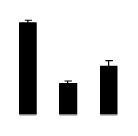
537	phosphoglycerate kinase	*R. pseudoacacia*	2257598	23.67/6.49	45.66/6.32	479	34	100	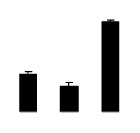
**Metabolism**									
498	putative plastidic glutamine synthetase	*O. sativa* (japonica group)	115461066	47.56/5.96	51.16/5.52	98	13	25	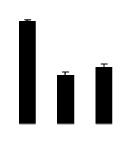
508	chalcone synthase	*R. pseudoacacia*	194740616	32.58/5.75	50.19/5.09	115	13	46	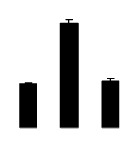
789	aconitate hydratase domain protein	*A. cellulolyticus* CD2	303239527	19.84/4.86	28.47/4.45	91	11	58	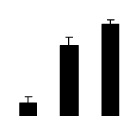
536	pyruvate kinase	*C. reinhardtii*	159485206	23.201/6.2	40.20/6.22	86	23	15	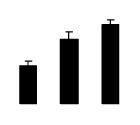
**Photosynthesis**									
506	Rubisco large subunit	*G. subaequalis*	24634972	52.37/6.34	50.37/5.13	215	31	45	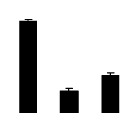
538	Rubisco large subunit	*Caesalpinia sp*. SH-2010	306481385	43.78/7.38	45.71/6.67	310	32	49	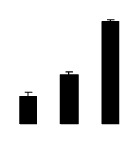
554	Rubisco large subunit	*Parkia multijuga*	148590322	51.78/6.23	34.27/6.41	190	19	27	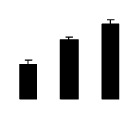
557	Rubisco large subunit	*Merremia hastata*	21634009	47.96/6.42	32.77/6.30	216	18	25	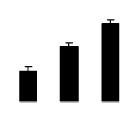
575	Rubisco large subunit	*Dendrobium aphyllum*	300250366	22.77/6.2	22.67/5.39	217	13	54	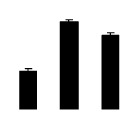
582	Rubisco large subunit	*Loxocarya gigas*	5737828	49.48/6.43	21.50/5.29	258	11	26	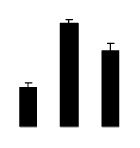
584	Rubisco large subunit	*Ipomoea purpurea*	157325538	53.35/6.41	37.47/6. 26	182	18	31	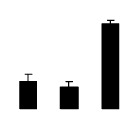
593	Rubisco large subunit	*Millettia lenneoides*	18032763	51.53/6.04	24.01/6.22	239	18	24	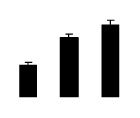
603	Rubisco large subunit	*R. pseudoacacia*	2343004	49.20/6.13	19.78/5.88	336	31	54	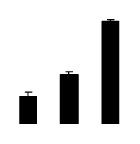
610	Rubisco large subunit	*Haematoxylum brasiletto*	66735773	18.40/6.05	17.95/5.17	179	14	46	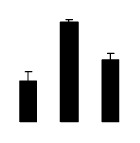
612	Rubisco large subunit	*Marila laxiflora*	49823207	21.44/5.91	18.190/4.98	282	12	35	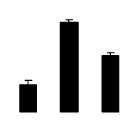
615	Rubisco large subunit	*R.pseudoacacia*	340511916	50.20/6.14	18.69/5.28	305	32	53	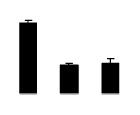
621	Rubisco large subunit	*Prunus salicina*	15987094	51.31/6.99	16.61/4.30	147	14	20	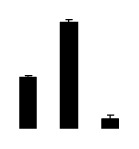
622	Rubisco large subunit	*Cecropia palmata*	6983898	52.00/6.23	16.72/4.54	126	13	16	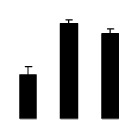
623	Rubisco large subunit	*R. pseudoacacia*	2342974	52.01/6.14	15.83/5.07	116	15	28	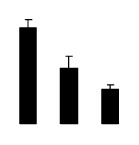
624	Rubisco large subunit	*Stigmaphyllon paralias*	14599610	52.24/6.23	15.23/4.41	175	21	31	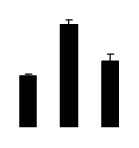
626	Rubisco large subunit	*Codonopsis dicentrifolia*	194400582	50.82/6.19	15.03/4.96	118	15	30	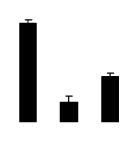
702	Rubisco large subunit	*Millettia lenneoides*	18032763	51.53/6.04	22.37/5.72	271	23	47	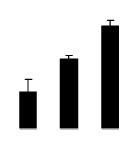
721	Rubisco large subunit	*Parthenocissus himalayana*	16973408	51.68/6.34	31.78/4.26	196	15	40	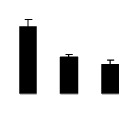
795	Rubisco large subunit	*R. pseudoacacia*	67079090	25.32/6.23	16.84/5.84	354	25	55	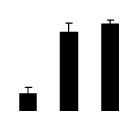
567	Ribulose-bisphosphate carboxylase	*Mangifera indica*	7261036	24.43/6.71	27.20/6.56	227	19	64	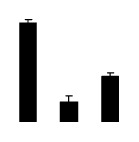
597	Ribulose-bisphosphate carboxylase	*Centrosema* sp. SH-2010	306481395	49.81/6.44	22794/6.23	167	18	27	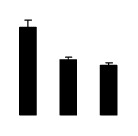
548	Ribulose-biphosphate carboxylase oxygenase	*Liparia genistoides*	146188483	27.28/6.36	37.36/6.06	263	25	60	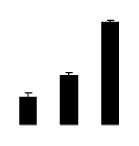
533	photosystem II protein 33kD	*Spinacia oleracea*	224916	26.65/5.01	38.28/5.34	294	22	78	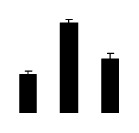
578	polypeptide of the oxygen evolving complex of photosystem II	*Sonneratia apetala*	146454492	24.99/5.61	24.59/5.03	95	6	26	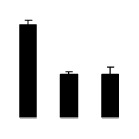
475	ATP synthase CF1 alpha subunit	*R. communis*	339516150	55.52/5.22	70.28/5.03	340	26	31	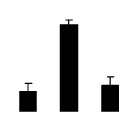
476	putative ATP synthase beta subunit	*O. sativa* (japonica group)	56784991	45.94/5.33	69.32/5.49	106	22	51	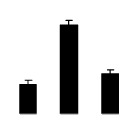
**Transportation**									
480	aspartyl/glutamyl-tRNA(asn/gln) amidotransferase subunit b	*Stigmatella aurantiaca* DW4/3-1	310819540	53.54/5.56	59.82/6.02	74	11	23	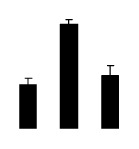
**Transcription/translation related**									
625	maturase-like protein	*Coursetia weberbaueri*	23664381	60.78/9.42	15.06/4.94	193	18	39	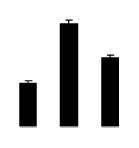
**Stress and defense**									
455	heat shock protein 70	*Cucumis sativus*	1143427	75.37/5.15	100.32/4.83	332	21	25	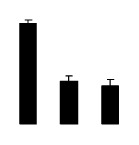
478	agglutinin I polypeptide B	*R. pseudoacacia*	4033451	31.19/6.14	64.35/5.50	117	14	69	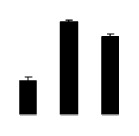
482	agglutinin I polypeptide B	*R. pseudoacacia*	4033451	31.19/6.14	60.65/6.19	488	19	69	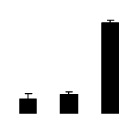
525	plastidic aldolase	*N. paniculata*	4827253	43.07/6.38	46.90/6.47	202	25	48	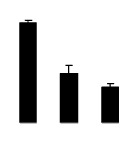
800	phenylalanine ammonia lyase	*R. pseudoacacia*	194740604	78.41/6.31	58.78/5.46	274	31	55	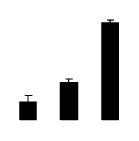
**Unclear classification**									
611	predicted protein	Micromonas sp. RCC299	171910308	31.90/5.47	18.68/5.22	280	31	62	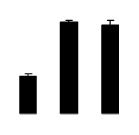

a Assigned spot number as indicated in Figure 9.

b Accession numbers according to the NCBIInr database.

c,d Theoretical (c) and experimental (d) masses (kDa) and pIs of identified proteins.

e Mascot protein score reported after searching against the NCBInr database. Experimental values were calculated by Image Master 2D Platinum software. Theoretical values were retrieved from the protein database.

f The number of unique peptides identified for each protein.

g Sequence coverage.

h Mean of relative protein abundance and standard error. Three treatments including 1, 5, and 10 days after 500 mM NaCl treatment were performed.

**Table 2:** Protein identities and their relative changes in leaves of tetraploid *Robinia pseudoacacia* under salt stress.

Spot No. a	Protein Name	Species	gi Number b	Theoretical MW(Da)/pI c	Experimental MW(Da)/pI d	Score e	M f	C (%) g	V% ± SE h (1/5/10 days)
**Energy**									
60	phosphoglycerate kinase	*N. benthamiana*	313585890	50.05/7.66	98.76/6.24	169	13	23	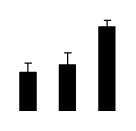
476	mitochondrial F1-ATPase beta subunit	*Dimocarpus longan*	269914683	59.87/6.18	22.47/4.62	274	23	38	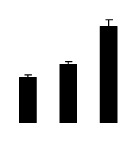
**Metabolism**									
229	chalcone synthase	*R. pseudoacacia*	194740620	36.76/6.22	27.61/6.24	608	29	32	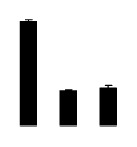
486	chalcone synthase	*R. pseudoacacia*	194740616	32.58/5.75	68.39/5.71	175	15	48	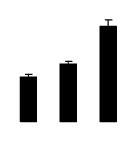
70	enolase	*Glycine max*	42521309	47.69/5.31	92.57/5.73	130	12	20	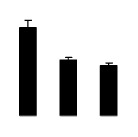
**Photosynthesis**									
79	Rubisco large subunit	*R. pseudoacacia*	340511916	50.71/6.14	98.81/6.59	299	34	62	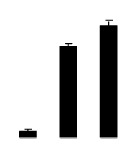
90	Rubisco large subunit	*Daviesia rhizomata*	18032753	51.67/6.14	67.60/6.28	418	37	63	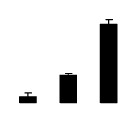
164	Rubisco large subunit	*Gironniera subaequalis*	24634972	52.37/6.34	44.69/6.22	340	35	47	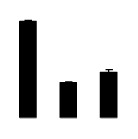
180	Rubisco large subunit	*Wisteria* sp.	2343020	51.49/6.13	39.61/6.19	294	20	32	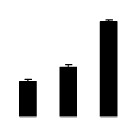
185	Rubisco large subunit	*Canavalia rosea*	18157259	52.11/6.14	39.79/6.24	209	17	30	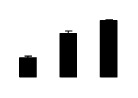
267	Rubisco large subunit	*R. pseudoacacia*	2343004	49.23/6.13	22.59/6.28	156	20	47	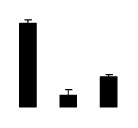
301	Rubisco large subunit	*Mascagnia stannea*	14599586	51.57/6.14	19.08/4.81	347	29	56	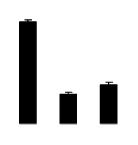
302	Rubisco large subunit	*Mascagnia stannea*	14599586	51.57/6.14	18.99/4.64	308	31	54	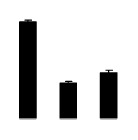
448	Rubisco large subunit	*Floerkea proserpinacoides*	38147280	51.57/5.87	24.53/4.89	120	14	23	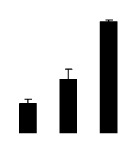
449	Rubisco large subunit	*Aspicarpa sericea*	331690047	49.79/6.13	22.68/4.81	175	16	27	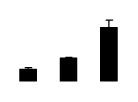
453	Rubisco large subunit	*Wisteria* sp.	2343020	51.49/6.13	27.71/6.32	311	26	42	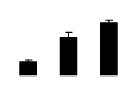
473	Rubisco large subunit	*Centrosema* sp. SH-2010	306481395	49.81/6.44	22.49/6.20	265	27	31	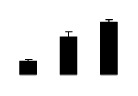
474	Rubisco large subunit	*Sassafras albidum*	283558279	20.64/6.05	21.07/6.24	132	14	32	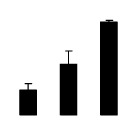
477	Rubisco large subunit	*Diospyros pentamera*	221078519	51.98/6.14	21.06/4.35	179	15	20	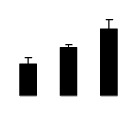
479	Rubisco large subunit	Mucuna macrocarpa	18157295	52.11/6.14	44.05/6.82	284	33	43	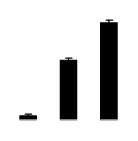
272	Rubisco large subunit	*Pachynema junceum*	9909908	19.07/5.33	20.07/5.68	226	14	36	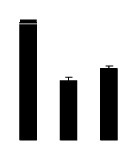
283	Rubisco large subunit	*Pachynema junceum*	9909908	19.19/5.33	19.14/5.18	199	14	36	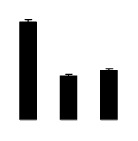
481	Rubisco activase	*Glycine max*	290766481	52.64/5.54	98.57/5.11	211	29	43	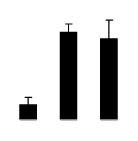
487	Rubisco activase	*Glycine max*	290766485	48.64/6.28	53.18/5.47	172	14	21	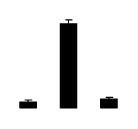
129	Rubisco activase	*Zantedeschia aethiopica*	13430334	37.25/6.7	55.76/6.03	157	18	35	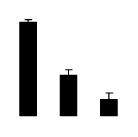
68	ATP synthase CF1 alpha subunit	*R. communis*	339516150	55.52/5.22	93.78/5.40	472	28	36	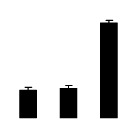
475	ATP synthase CF1 alpha subunit	*Vigna radiata*	289066833	55.68/5.21	21.06/6.24	366	28	35	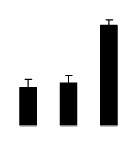
120	Phosphoribulokinase (PPK)	*Pisum sativum*	1885326	39.00/5.41	58.67/5.57	129	10	25	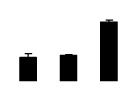
**Transportation**									
46	Rubisco subunit binding-protein beta subunit	*R. communis*	255564820	64.15/5.65	100.08/5.40	127	13	23	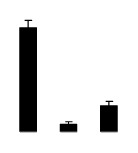
248	General secretion pathway protein D precursor, putative	*R. communis*	255619353	30.81/4.67	27.01/5.53	174	17	54	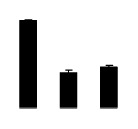
**Transcription/translation related**									
77	maturase-like protein	Olneya tesota	23477700	60.67/9.45	91.49/6.01	409	27	52	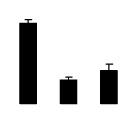
**Stress and defense**									
76	Chain A, legume lectin sf the bark of *robinia pseudoacacia*	*R. pseudoacacia*	15826665	25.58/4.48	99.77/6.42	121	13	57	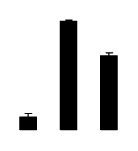
128	sedoheptulose-1,7-bisphosphatase(SBPase)	*Cucumis sativus*	229597543	42.08/5.96	55.09/5.22	99	11	23	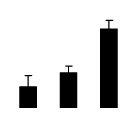
169	phenylalanine ammonia lyase	*R. pseudoacacia*	194740606	34.40/5.4	44.64/6.59	300	21	72	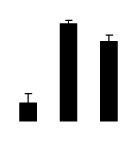
195	ascorbate peroxidase	*Medicago sativa*	16304410	20.14/5.33	32.49/5.26	110	6	27	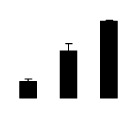
**Unclear classification**									
482	PREDICTED: ADP-ribosylation factor 1-like	*Amphimedon queenslandica*	340369230	20.47/6.15	49.38/5.26	89	2	4	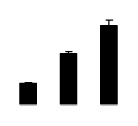
288	Em protein	*R. pseudoacacia*	1754977	12.22/6.21	28.51/5.30	356	22	98	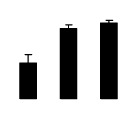

a Assigned spot number as indicated in Figure 10.

b Accession numbers according to NCBIInr database.

c,d Theoretical (c) and experimental (d) mass (kDa) and pI of identified proteins.

e Mascot protein score reported after searching against the NCBInr database. Experimental values were calculated by Image Master 2D Platinum Software. Theoretical values were retrieved from the protein database.

f The number of unique peptides identified for each protein.

g Sequence coverage.

h Mean of relative protein abundance and standard error. Three treatments including 1, 5 and 10 days after 500 mM NaCl treatment were performed.

## Abbreviations

APX: ascorbate peroxidase
BGR: the relative growth rate of stem basal diameter
BSA: bovine serum albumin
CHS: chalcone synthase
2-DE: Two-dimensional electrophoresis
HGR: The relative growth rate of height
H_2_O_2_: hydrogen peroxide
Hsp70: heat shock protein 70
MDA: Malondialdehyde
PGK: phosphoglycerate kinase
PPK: phosphoribulokinase
RGR: the relative growth rate of range
ROS: Reactive Oxygen Species
RuBPCase: ribulose-bisphosphate carboxylase
RWC: relative water content
SBPase: sedoheptulose-1,7-bisphosphatase
TCA: tricarboxylic acid

## References

[1] Horie T., Schroeder J.I. (2004). Sodium transporters in plants. Diverse genes and physiological functions. Plant Physiol.

[2] Zhu J.K. (2001). Plant salt tolerance. Trends Plant Sci.

[3] Srivastava A.K., Ramaswamy N.K., Mukopadhyaya R., Chiramal J.M.G., D’Souza S.F. (2009). Thiourea modulates the expression and activity profile of mtATPase under salinity stress in seeds of *Brassica juncea*. Ann. Bot.

[4] Yang A., Dai X., Zhang W.H. (2012). A R2R3-type MYB gene, *OsMYB2*, is involved in salt, cold, and dehydration tolerance in rice. J. Exp. Bot.

[5] Breyne P., Zabeau M (2001). Genome-wide expression analysis of plant cell cycle modulated genes. Curr. Opin. Plant Biol.

[6] Salekdeh G.H., Siopongco J., Wade L.J., Ghareyazie B., Bennett J (2002). Proteomic analysis of rice leaves during drought stress and recovery. Proteomics.

[7] Zhang H., Han B., Wang T., Chen S., Li H., Zhang Y., Dai S (2012). Mechanisms of plant salt response: Insights from proteomics. J. Proteome Res.

[8] Romero-Aranda R., Bondada B.R., Syvertsen J.P., Grosser J.W. (1997). Leaf characteristics and net gas exchange of diploid and autotetraploid citrus. Ann. Bot.

[9] Wang Q.L., Yu M.D., Lu C., Wu C.R., Jing C.R. (2011). Study on breeding and photosynthetic characteristics of new polyploidy variety for leaf and fruit-producing mulberry (*Morus* L). Sci. Agric. Sin.

[10] Masterson J (1994). Stomatal size in fossil plants: Evidence for polyploidy in majority of angiosperms. Science.

[11] Stupar R.M., Bhaskar P., Yandell B., Rensink W.A., Hart A.L., Ouyang S., Veilleux R.E., Busse J.S., Erhardt R.J., Buell C.R. (2007). Phenotypic and transcriptomic changes associated with potato autopolyploidization. Genetics.

[12] Riddle N.C., Jiang H., An L., Doerge R.W., Birchler J.A. (2010). Gene expression analysis at the intersection of ploidy and hybridity in maize. Theor. Appl. Genet.

[13] Ramsey J (2011). Polyploidy and ecological adaptation in wild yarrow. Proc. Natl. Acad. Sci. USA.

[14] Li F.Z., Ning X.M., Qiu X.M., Su C.F., Yao J.Q., Tian L.W. (2012). Genetic mapping of the dark brown fiber *Lc1* gene in tetraploid cotton. Sci. Agric. Sin.

[15] Allario T., Brumos J., Colmenero-Flores J.M., Tadeo F., Froelicher Y., Talon M., Navarro L., Ollitrault P., Morillon R (2013). Large changes in anatomy and physiology between diploid Rangpur lime (*Citrus limonia*) and its autotetraploid are not associated with large changes in leaf gene expression. J. Exp. Bot.

[16] Podda A., Checcucci G., Mouhaya W., Centeno D., Rofidal V., del Carratore R., Luro F., Morillon R., Ollitrault P., Maserti B.E. (2013). Salt-stress induced changes in the leaf proteome of diploid and tetraploid mandarins with contrasting Na^+^ and Cl^−^ accumulation behaviour. J. Plant Physiol.

[17] Beest M., le Roux J.J., Richardson D.M., Brysting A.K., Suda J., Kubešová M., Pyšek P. (2012). The more the better? The role of polyploidy in facilitating plant invasions. Ann. Bot.

[18] Lu C., Cui B., Huang L., Sun P., Zhang G., Li Y (2012). Phenotypic observation and analysis of inflorescence variation of Autotetraploid *Robinia pseudoacacia*. Sci. Silvae Sin.

[19] Al H.A., Monneveaux P., Nachit M.M. (1998). Direct and indirect selection for drought tolerance in alien tetraploid wheat durum wheat crosses. Euphytica.

[20] Fock I., Collonnier C., Purwito A., Luisetti J., Souvannavong V., Vedel F., Servaes A., Ambroise A., Kodja H., Ducreux G. (2000). Resistance to bacterial wilt in somatic hybrids between *Solanum tuberosum* and *Solanum phureja*. Plant Sci.

[21] Huang S., Sirikhachornkit A., Su X., Faris J., Gill B.S., Haselkorn R., Gornicki P (2002). Genes encoding plastid acetyl-CoA carboxylase and 3-phosphoglycerate kinase of the *Triticum/Aegilops* complex and the evolutionary history of polyploid wheat. Proc. Natl. Acad. Sci. USA.

[22] Zhang X.Y., Hu C.G., Yao J.L. (2010). Tetraploidization of diploid *Dioscorea* results in activation of the antioxidant defense system and increased heat tolerance. J. Plant Physiol.

[23] Xiong Y.C., Li F.M., Zhang T (2006). Performance of wheat crops with different chromosome ploidy: Root-sourced signals, drought tolerance, and yield performance. Planta.

[24] Meng F.J., Huang F.L. (2010). Changes of function and ultrastructure of mitochondria in *Robinia pseudoacacia* leaves under salt stress. Nonwood For. Res.

[25] Saleh B., Allario T., Dambier D., Ollitrault P., Morillon R (2008). Tetraploid citrus rootstocks are more tolerant to salt stress than diploid. C. R. Biol.

[26] Xiao X., Yang F., Zhang S., Korpelainen H., Li C (2009). Physiological and proteomic responses of two contrasting *Populus cathayana* populations to drought stress. Physiol. Plant.

[27] Verslues P.E., Agarwal M., Katiyar-Agarwal S., Zhu J., Zhu J.K. (2006). Methods and concepts in quantifying resistance to drought, salt and freezing, abiotic stresses that affect plant water status. Plant J.

[28] Apel K., Hirt H. (2004). Reactive oxygen species: Metabolism, oxidative stress, and signal transduction. Annu. Rev. Plant Biol.

[29] Tuna A.L., Kaya C., Dikilitas M., Higgs D (2008). The combined effects of gibberellid acid and salinity on some antioxidant enzyme activities, plant growth parameters and nutritional status in maize plants. Environ. Exp. Bot.

[30] Ashraf M (2009). Biotechnological approach of improving plant salt tolerance using antioxidants as markers. Biotechnol. Adv.

[31] Barhoumi Z., Djebali W., Chaïbi W., Abdelly C., Smaoui A (2007). Salt impact on photosynthesis and leaf ultrastructure of *Aeluropus littoralis*. J. Plant Res.

[32] Zapata J.M., Guéra A., Esteban-Carrasco A., Martín M., Sabater B (2005). Chloroplasts regulate leaf senescence: Delayed senescence in transgenic *ndhF*-defective tobacco. Cell Death Differ.

[33] Ishikawa T., Yoshimura K., Tamoi M., Takeda T., Shigeoka S (1997). Alternative mRNA splicing of 3′-terminal exons generates ascorbate peroxidase isoenzymes in spinach (*Spinacia oleracea*) chloroplasts. Biochem. J.

[34] Ndimba B.K., Chivasa S., Simon W.J., Slabas A.R. (2005). Identification of *Arabidopsis* salt and osmotic stress responsive proteins using two-dimensional difference gel electrophoresis and mass spectrometry. Proteomics.

[35] Portis J.A.R. (2003). Rubisco activase: Rubisco’s catalytic chaperone. Photosynth. Res.

[36] Parker R., Flowers T.J., Moore A.L., Harpham N.V.J. (2006). An accurate and reproducible method for proteome profiling of the effects of salt stress in the rice leaf lamina. J. Exp. Bot.

[37] Sudhir P.R., Pogoryelov D., Kovacs L., Garab G., Murthy S.D. (2005). The effects of salt stress on photosynthetic electron transport and thylakoid membrane proteins in the cyanobacterium *Spirulina platensis*. J. Biochem. Mol. Biol.

[38] Ma H., Song L., Shu Y., Wang S., Niu J., Wang Z., Yu T., Gu W., Ma H., Baker N.R. (2012). Comparative proteomic analysis of seedling leaves of different salt tolerant soybean genotypes. J. Proteomics.

[39] Davletova S., Rizhsky L., Liang H., Sheng A., Oliver D.J., Coutu J., Shulaev V., Schlauch K., Mittler R (2005). Cytosolic ascorbate peroxidase 1 is a central component of the reactive oxygen gene network of *Arabidopsisi*. Plant Cell.

[40] Shakirova F.M., Bezrukova M.V., Khairullin R.M. (1993). The increase in lectin level in wheat shoots under the action of salt stress. Izv. Russ. Acad. Sci.

[41] Kosová K., Vítámvás P., Prášil L.T., Renaut J (2011). Plant proteome changes under abiotic stress-contribution of proteomics studies to understanding plant stress response. J. Proteomics.

[42] Wolf B (1982). A comprehensive system of leaf analyses and its use fore diagnosing crop nutrient status. Commun. Soil Sci. Plant Anal.

[43] Chen S., Li J., Wang S., Hüttermann A., Altman A (2001). Salt, nutrient uptake and transport, and ABA of *Populus euphratica*; a hybrid in response to increasing soil NaCl. Trees.

[44] Roth E.F., Gilbert J.H.S. (1984). Pyrogallol assay for SOD: Absence of a glutathione artifact. Anal. Biochem.

[45] Nickel R.S., Cunningham B.A. (1969). Improved peroxidase assay method using leuco 2,3,6-trichloroindophenol and application to comparative measurements of peroxidase catalysis. Anal. Biochem.

[46] Nordhoff A., Bucheler U.S., Werner D., Schirmer R.H. (1993). Folding of the four domains and dimerization are impaired by the Gly446→Glu exchange in human glutathione reductase. implications for the design of antiparasitic drugs. Biochemistry.

[47] Nakano Y., Asad K (1981). Hydrogen peroxide is scavenged by ascorbate-specific peroxidase in spinach chloroplast. Plant Cell Physiol.

[48] Hurkman W.J., Tanaka C.K. (1986). Solubilization of plant membrane proteins for analysis by two-dimensional gel electrophoresis. Plant Physiol.

[49] Bradford M (1976). A rapid and sensitive method for the quantification of microgram quantities of protein utilizing the principle of protein-due binding. Ann. Biochem.

